# Psychiatric Assistance Dogs for Adults with Mental Health Conditions: Use, Perceived Effectiveness and Challenges

**DOI:** 10.3390/bs16071178

**Published:** 2026-07-13

**Authors:** Yuxuan Zhang, Amanda J. Salmon, Nancy A. Pachana

**Affiliations:** School of Psychology, The University of Queensland, Brisbane, QLD 4072, Australia; zhangyx3022@163.com (Y.Z.); a.salmon@uq.edu.au (A.J.S.)

**Keywords:** psychiatric assistance dogs, animal-assisted interventions, mental health, safety regulation, emotional regulation

## Abstract

Psychiatric assistance dogs (PADs) are increasingly recognised as complementary supports for individuals with mental health conditions, yet their role in everyday functioning as well as handler and animal welfare remains underexplored. This study explored how PADs are used by individuals with a range of mental health conditions, the benefits they provide, and any challenges experienced in terms of both handler and animal well-being. An online mixed-methods survey was conducted, yielding 85 valid responses. Quantitative data were analysed descriptively; qualitative responses were examined using Leximancer. Findings suggest that PADs provide benefits beyond specific trained tasks, offering ongoing support that helps handlers feel safer and more able to engage in activities of daily living and mental health treatment. At the same time, participants reported challenges such as stigma, public misunderstanding of public access rights, and financial costs, which can limit the effectiveness of PAD support. Many participants also reported relying heavily on their PAD, which may have detrimental consequences for the well-being of both handler and animal. These findings highlight the importance of improving public awareness, access, and support systems to maximise the benefits of PADs and help ensure the well-being and safety of both PADs and their handlers. Future research should consider both human and animal perspectives in greater depth to better understand these partnerships.

## 1. Introduction

According to the World Health Organisation (WHO), approximately 16% of the global population lives with some form of disability (WHO, 2022). Importantly, people with disabilities continue to experience substantial disadvantages in health care access and increased exposure to stigma, abuse, and neglect (WHO, 2022). This growing population underscores the need for research examining how different disability experiences affect quality of life and access to support within contemporary health and social systems. Among people living with disabilities, those who experience mental health conditions or intellectual impairments may experience greater disadvantage than those with physical or sensory impairments (WHO & World Bank, 2011). In Australia, as in other parts of the world, among various psychosocial supports, psychiatric assistance dogs (PADs) have been recognised as a promising intervention for individuals with mental health disabilities (Foltin & Glenk, 2023; Lloyd et al., 2019).

Like guide dogs and hearing dogs, PADs are legally recognised service dogs that receive protection under many jurisdictions, with access to public spaces typically off-limits to companion animals (Howell & Salmon, 2025). Unlike emotional support dogs (ESDs), PADs are trained to perform specific tasks that mitigate the symptoms of mental health disorders, such as anxiety, depression, or bipolar disorder (Rodriguez et al., 2020a). This distinction is particularly important, as public confusion surrounding different categories of assistance animals may contribute to questioning, stigma, and access barriers experienced by legitimate service dog handlers (McManus et al., 2021; Schoenfeld-Tacher & Kogan, 2017). The rapidly expanding number of terms used to describe animals in assistive roles has not only contributed to public confusion but may also hamper research efforts (Howell et al., 2022; Parenti et al., 2013).

Specific examples of tasks PADs perform include alerting handlers to signs of anxiety, manic or panic episodes, and interrupting suicidal ideation, self-harming, or compulsive actions. While ESDs have no public access rights protected by Australian legislation, PADs are permitted to access public areas after the completion of a certification and Public Access Test (PAT) process (Lloyd et al., 2019). These policy supports and legal recognitions align with growing attention to the limitations of conventional treatments for some individuals with mental health issues (e.g., Rymaszewska et al., 2023).

In response to the limitations of conventional treatments for some mental health conditions, PADs have gained increasing attention globally as a novel, complementary, and integrative therapeutic approach, although their implementation is not without challenges (Foltin & Glenk, 2023). However, research examining their use among individuals with mental health disabilities remains limited. Much of the existing research has focused on PADs assisting military veterans with post-traumatic stress disorder (PTSD; Hansen et al., 2023), with studies demonstrating significant improvements in treatment outcomes (e.g., O’Haire & Rodriguez, 2018; Rodriguez et al., 2020b). Nevertheless, this focus overlooks the diverse needs of the broader population with mental health conditions. As a result, little is known about the applicability of PADs across more general mental health contexts, including the implications for both handlers and the dogs themselves.

Although several studies have examined the challenges faced by assistance dogs in general, such as negative public attitudes and welfare concerns related to intensive training (e.g., Salmon et al., 2022), little research has specifically addressed the issues encountered in the use of PADs. For example, PAD handlers may encounter variability in their satisfaction with training, which can undermine the therapeutic effectiveness of the human–animal dyad (Lloyd et al., 2019). In addition, individual factors among PAD handlers such as age, gender, psychiatric diagnosis, level of functional impairment, and duration of partnership with the dog may influence how support is delivered, experienced, and interpreted (Lloyd et al., 2019). Understanding these factors is crucial for identifying the patterns of PAD use, improving support delivery, and enhancing the perceived effectiveness of PADs in managing psychiatric conditions.

Given the relative paucity of research on the use of PADs in more general mental health contexts, this exploratory study aimed to examine how PADs are used and experienced by individuals with diverse mental health conditions. In particular, this study sought to explore patterns of PAD use in everyday life, how individual characteristics of handlers are associated with perceived effectiveness, and how PADs are experienced as complementary supports in relation to safety, emotional regulation, daily functioning, and engagement with treatment. The study also examined challenges and barriers associated with PAD use, including issues related to training, public access, stigma, reliance on the animal, and ongoing maintenance requirements. By adopting this perspective, the study further considers how PADs may function as ongoing, embedded supports within broader systems of care and contributes to a more integrated understanding of PADs as relational supports.

## 2. Materials and Methods

### 2.1. Participant Characteristics

A total of 103 participants who lived with and were supported by a certified PAD took part in the current study. All participants were recruited through the Australian mindDog Facebook forum and were required to be over 18 years old, with their PADs having passed the PAT successfully. All participants who submitted a survey were eligible for the prize draw (one of two AUD$50 gift vouchers) regardless of whether their data were included in the final analyses.

### 2.2. Procedures

This exploratory study was conducted according to the National Statement on Ethical Conduct in Human Research and was reviewed by the Human Research Ethics Committees (HRECs) at BLINDED (Project Identifier: 2025/HE000487). A survey link generated using an online platform (Qualtrics-XM) was distributed through the mindDog Facebook forum. Participating in the survey was considered to indicate informed consent. Participation was voluntary, and all participants could withdraw from the survey at any time without penalty. Details for support services, if required, were provided in the participant information sheet. The recruitment window was approximately 2 months (June–July 2025).

### 2.3. Measures

This study used a self-developed questionnaire designed specifically to examine PAD handlers’ demographic characteristics, mental health conditions, modes and extent of PAD use, and experiences of handler training. Item development was guided in part by an accredited PAD assessor and a certified PAD trainer to ensure relevance to real-world PAD use and training.

The questionnaire consisted of 16 items, including both closed- and open-ended questions. All demographic characteristics (age, gender, education level, PAD use duration, and employment status) and mental health and treatment variables (current mental health conditions and treatment status) were assessed using nine closed-ended multiple-choice questions. Examples of the closed-ended questions include “Has your dog passed the PAT (Public Access Test)?” and “Are you currently receiving treatment or therapy for your mental health condition(s)?” Modes of PAD use were explored using six multiple-choice, multiple-response, and open-ended questions. Finally, participants provided open-ended responses describing their experiences with PAD training.

### 2.4. Analysis

#### 2.4.1. Data Preparation

All responses were checked for completeness and validity. Responses with less than 85% completion were excluded, resulting in a final sample of 85 participants. Responses to “Other (please specify)” options were reviewed and re-coded into existing categories when possible.

#### 2.4.2. Data Analyses

Quantitative data were analysed using Microsoft Excel, as the analyses were limited to descriptive statistics (frequencies, percentages, means, and standard deviations), appropriate for the exploratory aims and sample size of the study. Descriptive statistics were calculated for closed-ended items.

Open-ended responses were analysed using a qualitative descriptive approach, informed by principles of thematic analysis and mixed-methods integration. This approach was selected because the study aimed to summarise participants’ experiences and perceived meanings in relation to PAD use, rather than to develop a new theory or test an a priori explanatory model (Sandelowski, 2000, 2010). Leximancer (Version 5.0; Leximancer Pty Ltd., 2018, Brisbane, Australia) was used as a computer-assisted text analysis tool to identify patterns of word co-occurrence and relationships between concepts across the open-ended responses. In this study, Leximancer was used as an analytic aid rather than a substitute for researcher interpretation. The concept maps and concept lists provided a structured starting point for identifying salient patterns in the text, but the final themes reported in the Results section were developed through researcher review and interpretation of the original participant responses.

Prior to analysis, text responses were checked for clarity and logical consistency. Responses that repeated content from earlier closed-ended questions but still reflected meaningful information (e.g., indicating reliance on the PAD itself as the only strategy) were retained. By contrast, logically inconsistent responses were removed, and their corresponding follow-up text answers were excluded.

The qualitative analytic process involved several steps. First, responses to each open-ended item were read in full to support familiarisation with the content and context of participants’ accounts. Second, Leximancer outputs were generated separately for each relevant open-ended question, including concept maps and ranked concept lists. Third, these outputs were reviewed alongside the original responses to examine how software-identified concepts were being used by participants in context. Concepts that were closely related in meaning, or that reflected similar experiences across responses, were grouped into broader researcher-interpreted themes. Where the automated Leximancer labels were too narrow, ambiguous, or insufficiently descriptive, theme labels were revised to better reflect the meaning of participants’ accounts. This was in line with thematic analysis principles, in which themes are developed through active researcher interpretation rather than simply discovered within the data (Braun & Clarke, 2006). Representative quotations were then selected to illustrate each theme and to check that the interpretation remained grounded in the data.

In Leximancer concept maps, themes are represented by coloured circles whose size and proximity reflect their relative prominence and co-occurrence within the text (Leximancer Pty Ltd., 2018). For the revised manuscript, the original Leximancer maps were adapted to improve readability and to more clearly communicate the researcher-interpreted findings. The adapted figures retain the broad relationships identified in the Leximancer outputs, including relative prominence and conceptual overlap, while presenting clearer theme labels and supporting concepts derived from researcher interpretation of the maps and participant responses.

#### 2.4.3. Integrated Analysis

For paired questions, quantitative and qualitative data were analysed together using an integrated mixed-methods approach (Fetters et al., 2013). Descriptive statistics from the closed-ended questions were integrated with themes from the corresponding open-ended responses, allowing both numerical findings and qualitative results to be presented in a complementary way. Integration occurred during interpretation and reporting rather than through statistical transformation of the qualitative data. For example, quantitative findings on participants’ reported safe time away from their PAD were interpreted alongside qualitative accounts of the strategies participants used when separated from their dog. Similarly, descriptive findings concerning key contexts of PAD support and challenges of PAD use were interpreted alongside qualitative themes relating to safety, emotional regulation, public stigma, access barriers, and practical support needs. This mixed-methods integration enabled the identification of core experiential themes, including feelings of safety, emotional support, impacts on daily functioning, and perceived effectiveness of PAD training, which were further interpreted in relation to complementary treatment frameworks.

## 3. Results

### 3.1. Participant Characteristics

A total of 85 participants met inclusion criteria and completed at least 85% of the survey. The majority of the sample were female (81.2%), with a smaller proportion of males (12.9%) or non-binary/third gender (5.9%). The age of participants ranged from 19 to 88 years, with a mean of 50.07 (SD = 15.52). Over half of the sample had completed a degree (51.8%), followed by completion of some university/technical school (21.2%). Other participants reported high school or equivalent (12.9%) or vocational/technical school (10.6%). Three participants (3.6%) did not indicate educational level.

Employment status varied. The largest group (37.7%) reported they were not able to work due to disability, followed by participants employed full-time (20.0%), retired (15.3%), self-employed (12.9%), employed part-time (10.6%), or a student (3.5%). In this sample, most of the participants indicated that they had more than one year of PAD use (68.2%), while 24.7% reported 2–12 months of PAD use. Only a few participants (7.1%) reported PAD use for less than one month. The average duration of PAD use was 40.15 months (SD = 39.27, range = 1–216).

### 3.2. Mental Health and Treatment

A wide range of mental health conditions were reported, and respondents could report more than one mental health condition. The most frequently reported conditions were PTSD (post-traumatic stress disorder; 26.3%) and anxiety disorders like generalised anxiety disorder, social anxiety, and panic disorder (25.0%). Another commonly reported condition was ADHD or other neurodevelopmental disorders (20.4%), followed by depression (14.5%). Less frequently reported conditions included eating disorders and schizophrenia or other psychotic disorders. With respect to current treatment status, most participants (43.5%) reported receiving both therapy and medication, with 36.3% of participants reporting either using medication or being engaged in therapy but not both.

### 3.3. PAD Use Mode Characteristics

**Figure 1:**
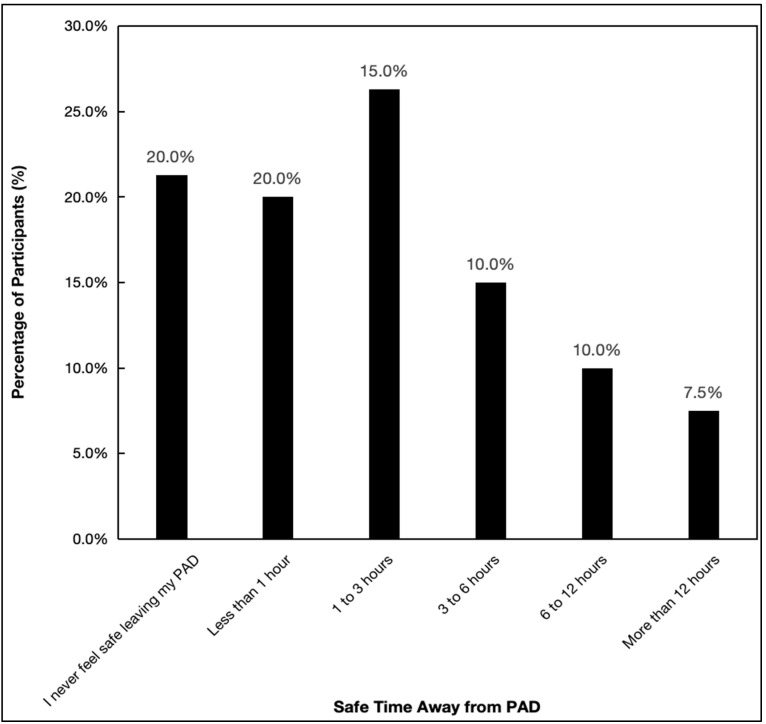
Distribution of Participants Reported Safe Time Away from Their PAD. Note. Percentages may not total 100 because of rounding.

Overall descriptive patterns indicated that PADs were most frequently relied upon in public and high-stress situations, with safety and emotional regulation emerging as central functions across use contexts. As presented in Figure 1, participants reported varied levels of comfort when separated from their PADs. The largest group (26.3%) indicated they felt safe being apart for one to three hours. A notable group (21.3%) stated they never felt safe without their PAD, followed by groups that reported shorter safe periods of less than one hour (20.0%) or longer durations exceeding three hours but less than six hours (15.0%). A much smaller group (10%) of participants indicated that they were comfortable being away from their PADs for a duration of six to 12 h. Only 7.5% of participants reported feeling comfortable being away from their PADs for more than 12 h. These results suggest that while some participants can manage limited time without their PADs, many experience strong reliance and potential distress in their absence. This was the most notable finding in the survey, with implications for the welfare of both human handlers and the PADs themselves.

**Figure 2:**
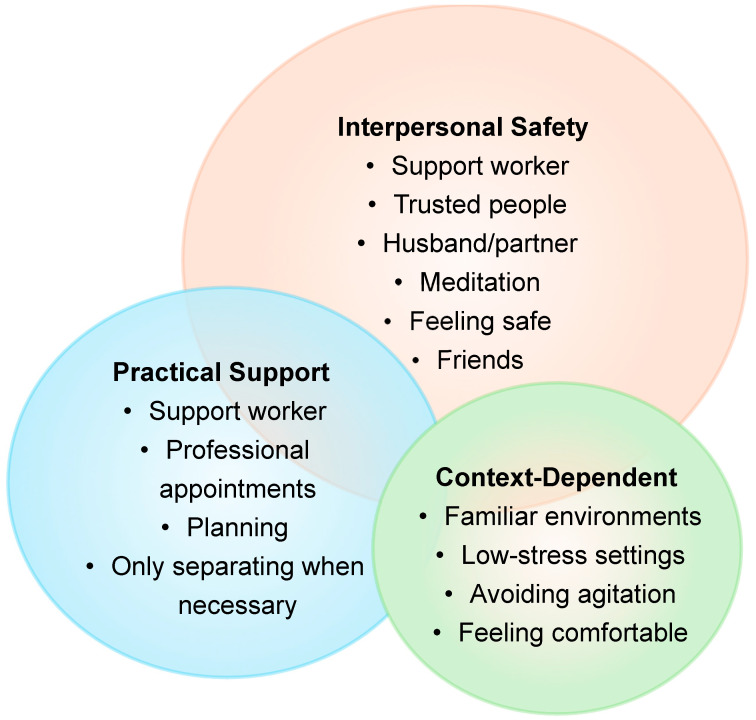
Concept Map of Strategies Used When Away from the PAD. Note. This figure was adapted from Leximancer outputs to enhance readability and interpretive clarity. Circle size represents relative theme prominence and overlap between circles indicates conceptual co-occurrence between themes. Theme labels and supporting concepts were refined by the research team through interpretation of the Leximancer outputs and participant responses.

The 76 participants described a range of strategies they used when separated from their PADs. Three researcher-interpreted themes were identified: interpersonal safety, practical support, and context-dependent separation. These themes were not mutually exclusive but instead reflected overlapping strategies that participants used to manage time away from their PAD. Relative theme prominence is shown in Figure 2 through circle size, while conceptual overlap is shown through overlapping circles.

#### 3.3.1. Theme 1: Safety Through Continuous Presence

The most prominent theme was interpersonal safety. This theme reflected participants’ reliance on trusted people or familiar interpersonal supports when separated from their PAD. Participants described feeling more able to manage time away from their dog when they were with people who knew them, understood their needs, or could provide reassurance and assistance if distress occurred. Within this theme, support workers, trusted people, husbands or partners, friends, and strategies such as meditation were linked to feeling safe. Representative quotes included:

“Meditation and activity distracting myself.”

(Participant 74)

“I make sure I am with people who know me or I’m going to attend a professional appointment i.e., GP, Psychologist etc.”

(Participant 14)

#### 3.3.2. Theme 2: Functional Support in Daily Life

The second theme was practical support. This theme referred to external supports and planned arrangements that made separation from the PAD more manageable. Participants described using support workers, professional appointments, planning, or only separating from the PAD when necessary. In this theme, separation was not experienced as simply a matter of individual coping but as something that often required practical preparation or the presence of another support structure. Representative quotes included:

“Have a support worker with me.”

(Participant 33)

“Set clear objectives/tasks and only separating when necessary.”

(Participant 09)

#### 3.3.3. Theme 3: Negotiating Separation from PADs

The third theme was context-dependent separation. This theme captured the extent to which participants’ ability to be away from their PAD depended on the surrounding environment. Separation was more manageable in familiar, predictable, or low-stress settings, whereas unfamiliar, crowded, or emotionally demanding environments made separation more difficult or unacceptable for some participants. Representative quotes included:

“Being familiar with the environment prior to going without my PAD.”

(Participant 52)

“Only go somewhere that does not make me feel agitated.”

(Participant 62)

“I’m very rarely without her.”

(Participant 68)

Together, these responses, as shown below in Figure 2, indicated that coping strategies ranged from interpersonal and external supports to individual regulation, with some participants emphasising that their capacity to separate was conditional on the surrounding context or that they in fact never felt safe about separation. Overall, the qualitative findings extend the quantitative results by showing that time away from the PAD was not only a matter of duration but also depended on whether participants had adequate interpersonal, practical, and contextual supports in place.

### 3.4. Key Situations of PAD Support

**Figure 3:**
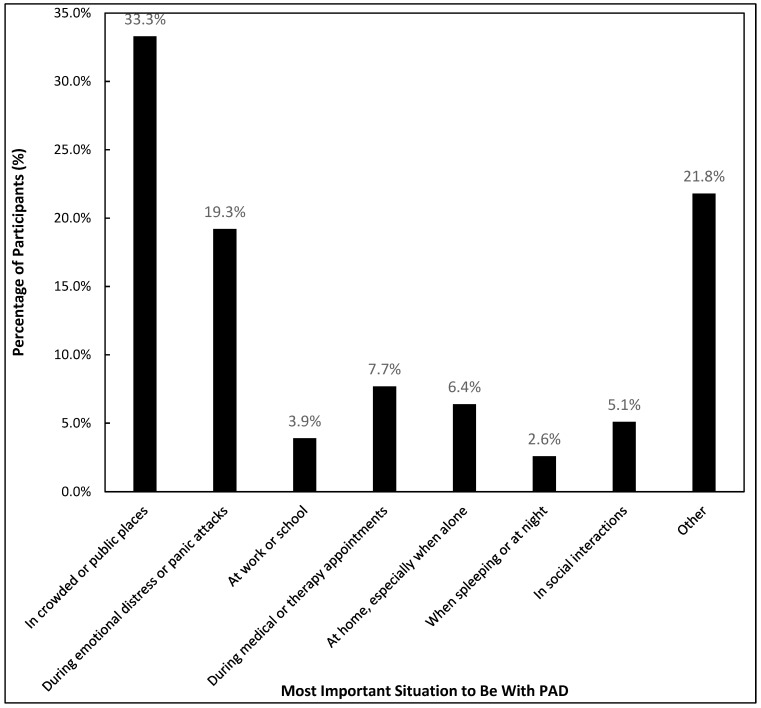
Most Important Situations in Which Participants Reported Needing Their PAD. Note. Percentages may not total 100 because of rounding. “Other” responses (n = 17) included “All of the above” (n = 11), “Always with my PAD” (n = 5), and “Except at home alone and sleeping” (n = 1).

Quantitative findings indicated that participants most frequently relied on PAD support in public and high-stress situations. As shown in Figure 3, participants most frequently identified crowded or public places (33.3%) and situations involving emotional distress or panic attacks (19.3%) as the most important contexts in which they required their PADs. Other contexts included medical or therapy appointments (7.7%), simply being at home (especially when alone; 6.4%), social interactions (5.1%), and work or school settings (3.9%). A smaller proportion indicated the most important context was when sleeping or at night (2.6%). In addition, a substantial proportion (21.8%) selected “other,” often specifying that all contexts were equally important or they were always with their PADs. Only one participant specified that all contexts were important except being at home alone or sleeping. These findings suggested that PADs were most valued for their support in highly emotionally demanding environments, while some participants perceived their PADs as generally necessary across daily life.

**Figure 4:**
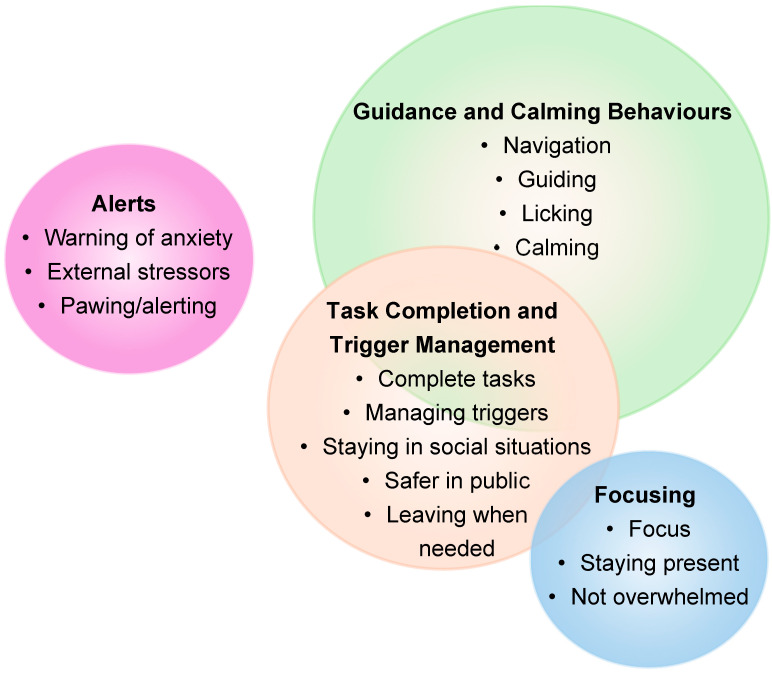
Concept Map of Perceived PAD Support in Key Situations in Crowded or Public Places. Note. This figure was adapted from Leximancer outputs to enhance readability and interpretive clarity. Circle size represents relative theme prominence and overlap between circles indicates conceptual co-occurrence between themes. The alerts theme is presented as a distinct concept because it appeared separately from the other themes in the Leximancer output. Theme labels and supporting concepts were refined by the research team through interpretation of the Leximancer outputs and participant responses.

Qualitative analysis further elaborated these patterns, highlighting themes related to safety and emotional regulation (Figure 4). Among the 61 participants who provided open-ended responses about PAD support in key situations, four researcher-interpreted themes were identified: task completion and trigger management, guidance and calming behaviours, focus, and alerts. These themes clarified how PADs supported participants in crowded or public places, where participants often described needing assistance to remain safe, regulated, and able to complete everyday activities.

#### 3.4.1. Theme 1: Managing Triggers

The most prominent theme was task completion and trigger management. This theme captured the role of PADs in helping participants remain in public settings long enough to complete necessary activities, such as shopping or attending social situations, while also managing distress or triggers. The meaning of this theme was functional as well as emotional: PADs were described as helping participants tolerate environments that might otherwise feel unsafe, overwhelming, or likely to trigger panic. Representative quotes included:

“Allows me to complete shopping, stay in a social situation without needing to leave too quickly.”

(Participant 35)

“He has specific tasks to help with sensory grounding, or to indicate to me to leave a situation that is triggering panic… He knows when it’s time to get up or go to sleep, when to eat meals, when I’ve been sitting still too long. He keeps me moving through the day.”

(Participant 29)

#### 3.4.2. Theme 2: Emotional Regulation

The second theme was guidance and calming behaviours. This theme reflected the ways PADs used trained or familiar behaviours to help participants navigate public environments and regulate distress. Participants described behaviours such as navigation, guiding, licking, and calming as helping them shift attention away from surrounding stimuli and back toward the dog. These behaviours appeared to support grounding and emotional regulation in situations where participants felt overstimulated or unsafe. A representative quote included:

“Licking does draw my attention to her instead of the surrounding.”

(Participant 74)

#### 3.4.3. Theme 3: Attention and Focus

A smaller theme was focus. This theme referred to the PAD’s role in helping participants remain present and less overwhelmed in public places. Although less prominent than task completion and guidance behaviours, this theme highlighted the attentional function of PAD support, particularly in contexts where external stimuli could become overwhelming. A representative quote included:

“She helps me to focus on us and be more present. Also, she protects me.”

(Participant 16)

#### 3.4.4. Theme 4: Anticipating Risk

Finally, alerts emerged as a distinct theme. Unlike the other themes, alerts appeared separately in the Leximancer output, suggesting that participants described alerting as a specific and recognisable form of PAD support. This theme referred to PAD behaviours that warned participants of increasing anxiety, emotional overwhelm, or external stressors, allowing them to respond before distress escalated. A representative quote included:

“He provides support by pawing at my leg, which alerts me to becoming overwhelmed… This helps me calm down and either be able to continue with shopping or whatever we were doing, or leave and go home.”

(Participant 14)

Together, these themes indicate that PAD support in crowded or public places was not limited to one form of assistance. Rather, participants described PADs as supporting public participation through a combination of task-based assistance, grounding and calming behaviours, attentional focus, and alerting to distress. These qualitative findings help explain why crowded or public places were the most frequently reported key context for PAD support in the quantitative results.

### 3.5. Challenges of PAD Use and Associated Impacts

**Figure 5:**
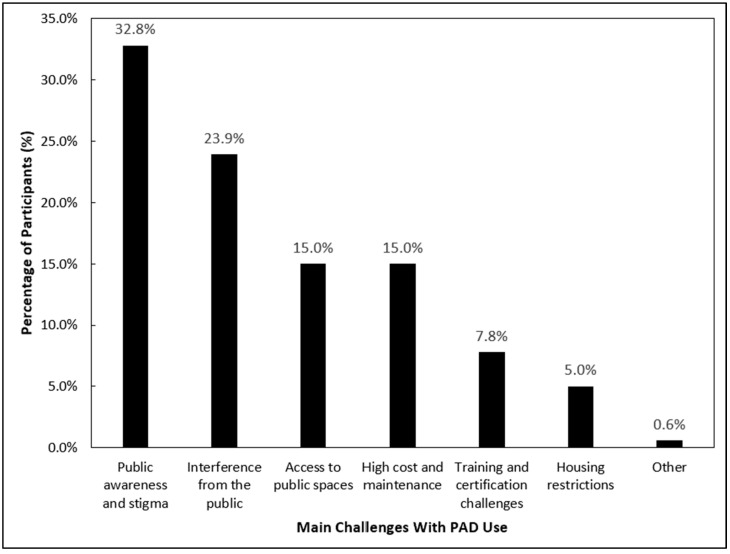
Challenges Reported When Using a PAD. Note. Percentages may not total 100 because participants could select multiple responses and because of rounding.

As shown in Figure 5, participants reported various challenges when using their PADs. The most frequently reported were stigma and lack of public awareness (32.8%), followed by interference from the public (23.9%). Financial cost and ongoing maintenance were also frequently mentioned (15.0%), along with restrictions on access to public spaces (15.0%). Smaller proportions reported difficulties with training and certification (7.8%) or housing restrictions (5.0%). One participant (0.6%) selected “other” but did not elaborate. These findings indicated that while stigma and social misunderstanding were the most common barriers, practical issues such as financial and access constraints also contributed to the challenges of PAD use.

**Figure 6:**
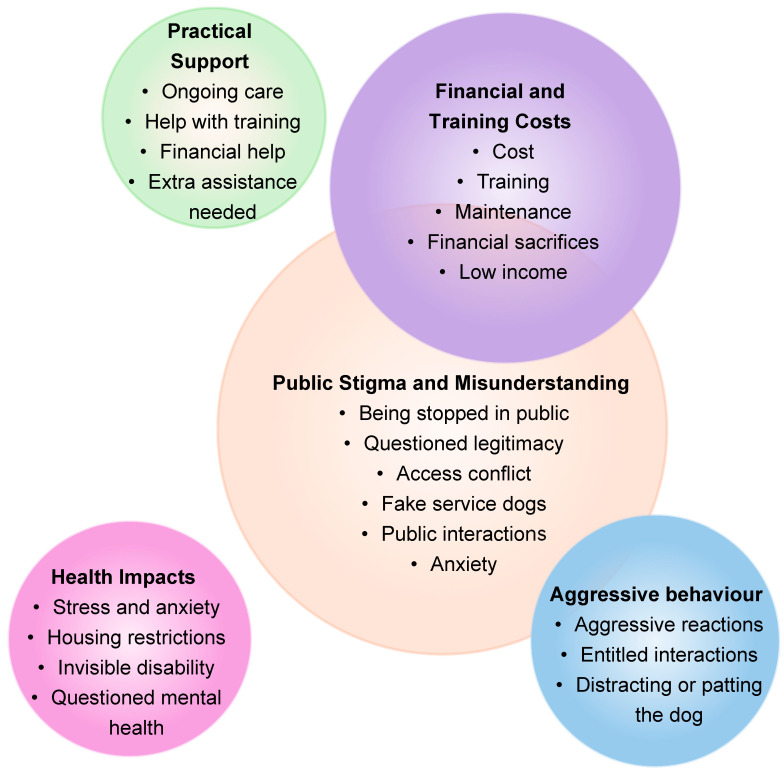
Concept Map of Impacts of Challenges Reported by Participants. Note. This figure was adapted from Leximancer outputs to enhance readability and interpretive clarity. Circle size represents relative theme prominence and overlap between circles indicates conceptual co-occurrence between themes. Theme labels and supporting concepts were refined by the research team through interpretation of the Leximancer outputs and participant responses.

Of the total sample (N = 85), 71 participants described a range of challenges they faced in living with or relying on their PADs. Five researcher-interpreted themes were identified: public stigma and misunderstanding, financial and training costs, practical support, aggressive behaviour, and health impacts. The relative prominence and overlap of these themes are shown in Figure 6. Consistent with the quantitative findings, public stigma and misunderstanding was the most prominent theme, while the remaining themes reflected practical, financial, interpersonal, and emotional consequences of PAD use.

#### 3.5.1. Theme 1: Stigma and Public Misunderstanding

The most prominent theme was public stigma and misunderstanding. This theme captured the social burden associated with using a PAD in public, particularly being stopped, questioned, challenged, or treated as though the PAD was not legitimate. Participants described repeated public interactions that required them to justify their dog’s presence, manage assumptions about fake service dogs, or decide whether to challenge access refusals. The meaning of this theme extended beyond inconvenience; participants described these encounters as increasing anxiety and reducing their willingness or ability to use their PAD in public. Representative quotes included:

“I use her less because of the constant conflict of being stopped from going places and accessing transport.”

(Participant 16)

“People assume his credentials are fake because he is not a Labrador. I am always ready to justify his training and presence but it is also common for me to just leave and not push access rights.”

(Participant 30)

“… Specially with all the fake/internet assistance dogs out there now. Which makes me more fearful of going out in public.”

(Participant 25)

“Being denied entry into shops etc can be so debilitating and causes severe anxiety.”

(Participant 64)

#### 3.5.2. Theme 2: Economic Burden

A second theme was financial and training costs. This theme reflected the expense and ongoing effort required to train, maintain, and care for a PAD. Participants described costs relating to training, veterinary care, grooming, food, insurance, and ongoing maintenance. For some, these costs required financial sacrifices and were particularly difficult in the context of low income, disability, or limited capacity to work. Representative quotes included:

“… I have pet insurance which is also expensive because I’m terrified I cannot pay for vet bills. Annual vaccines and checkups are expensive, food is expensive on the pension…”

(Participant 46)

“I struggle to get motivation to train him and take him out.”

(Participant 26)

“I worry that I may be unable to afford ongoing vet costs as he gets older and he requires grooming which I try to do myself due to my low income…”

(Participant 82)

#### 3.5.3. Theme 3: Environmental Challenges

The practical support theme reflected the ongoing care, assistance, and planning required to sustain PAD use. Although related to financial and training costs, this theme was broader than expense alone. It captured participants’ need for help with training, financial support, and extra assistance when their own health, energy, or resources were limited. This theme showed that PAD partnerships require ongoing practical support beyond initial certification or training. Representative quotes included:

“I prioritise money to his needs and training and make financial sacrifices…”

(Participant 69)

“… I need help from [my] children to assist training and financially.”

(Participant 76)

“… People feel they are entitled to know why I have him and what is wrong with me.”

(Participant 69)

#### 3.5.4. Theme 4: Risks to Dogs and Handlers

Aggressive behaviour was a further theme and reflected inappropriate or hostile responses from others. Participants described members of the public distracting, touching, patting, or trying to interact with the dog despite the dog’s working role. Some also described aggressive or entitled reactions when boundaries were set. This theme illustrated how public interference could undermine the PAD’s work and increase stress for the handler. Representative quotes included:

“… people also constantly try to distract the dog by making noises or trying to pat him when you are not looking like they have the right to do so…”

(Participant 15)

“I wish people didn’t see dogs as objects available to them for their attention or comments. I can’t imagine a person just touching a random person because they think they’re cute or loudly talking about someone as they pass by, but they think it’s ok with an assistance dog…”

(Participant 34)

#### 3.5.5. Theme 5: Health Impacts

Finally, the health impacts theme captured the psychological and emotional consequences of PAD-related challenges. Participants described stress and anxiety arising from public access issues, housing restrictions, invisible disability, and being questioned about their mental health. This theme reflected the way external barriers could directly affect participants’ well-being, particularly when PAD legitimacy or need was challenged by others. A representative quote included:

“The housing element has taken a large toll on my mental health. Whilst rentals must allow assistance animals often rentals can’t be obtained and the reasoning is “blamed” on other irrelevant factors.”

(Participant 09)

Together, as shown below in Figure 6, these findings reinforced the quantitative patterns of reported challenges while providing richer detail on their impacts, including the emotional toll of stigma, the practical burdens of training and maintenance, the questioning of handlers’ legitimacy, and the unique difficulties associated with invisible mental health conditions. Overall, the qualitative responses indicated that challenges related to PAD use extended beyond practical barriers to affect handlers’ psychological well-being and social experiences. In this sense, the qualitative findings show that the most prevalent reported challenges were not isolated practical problems but interconnected social, financial, and emotional barriers that shaped how effectively participants could use their PADs.

### 3.6. Perceived PAD Training Effectiveness

Of the total sample (N = 85), 68 participants provided open-ended feedback on the effectiveness of PAD training. Responses included both positive evaluations of training outcomes and critical reflections on its limitations, particularly regarding broader social issues. Two main themes were identified: training as practical and supportive, and training as limited in addressing systemic barriers. Unlike the previous qualitative sections, participant counts were available for these two broad response patterns. Most participants who responded to this item (n = 55) described PAD training as helpful or practical, while a smaller group (n = 13) indicated that training alone was not sufficient to address broader social, legal, or access-related barriers.

#### 3.6.1. Theme 1: Training Enables Access

Most participants (n = 55) emphasised that training had been helpful and practical, particularly in supporting their PADs to remain calm and enabling participants to respond appropriately in different situations. Responses highlighted that guidance from trainers and exposure to specific situations improved the PAD’s ability to provide support and that training offered detailed skills that equipped both PADs and handlers for everyday challenges. Also, training helped PADs to ignore distractions from the public. Some participants also specifically referred to mindDog, noting appreciation for the programme’s structure and support. Additionally, some participants also recognised the contribution made by the organisation and trainers. This theme suggested that training was valued not only for teaching the dog specific behaviours but also for helping the handler-dog dyad manage everyday situations more confidently. Representative quotes included:

“My trainer was very helpful in modelling ways to respond.”

(Participant 44)

“We are given strategies to deal with these situations but when we are unable we always have support from minddog and our trainers.”

(Participant 64)

“I feel my organisation mindDog does tremendous work in creating public awareness and working on removing stigma.”

(Participant 21)

#### 3.6.2. Theme 2: Systemic Barriers Remain

In contrast, 13 participants expressed concerns that the training alone was not enough. These responses reflected frustration that training could not fully address the issues such as public stigma. Some participants suggested that specific changes in society or legal reforms are needed regarding the use of PADs. This theme indicated that even effective PAD training may have limited impact when handlers continue to encounter social misunderstanding, discrimination, or inconsistent recognition of access rights. Representative quotes included:

“… greater public awareness and better social norms would be appreciated.”

(Participant 05)

“It’s systematic discrimination. Training does nothing. It requires legal change.”

(Participant 41)

Together, these findings indicated that while PAD training was widely valued for improving practical skills and support, participants also recognised its limits in overcoming systemic barriers rooted in social attitudes and discrimination. Thus, perceived training effectiveness appeared to depend not only on the quality of the training itself but also on whether broader public and institutional environments allowed the trained PAD-handler partnership to function as intended.

## 4. Discussion

This study examined patterns of PAD use among individuals living with mental health disabilities. The findings indicate that PAD use is highly individualised and embedded within broader social and support systems. The results further suggest that PADs function as ongoing supports, sometimes among the most critical and relied upon forms of support, enabling participation and emotional regulation in everyday environments. Across the qualitative findings, participants’ accounts were organised around overlapping themes of interpersonal and practical support, context-dependent safety, task completion, emotional regulation, public stigma, and practical burden. Rather than representing a narrowly defined intervention group, PAD use in the present sample was situated within diverse and complex life circumstances.

Handlers spanned the adult lifespan and reported varied employment situations and financial stability, indicating that PAD partnerships operate within broader social and functional challenges rather than isolated clinical conditions. Participants also described a wide range of mental health issues. Taken together, this diversity indicates that the benefits reported by handlers are unlikely to reflect condition-specific effects. Instead, the findings are consistent with PADs functioning as flexible supports that respond to shared experiences across different mental health presentations, particularly difficulties related to everyday functioning, environmental demands, and emotional regulation. This finding is supported by recent research on the use of psychiatric assistance animals to assist everyday functioning in the face of mental health challenges (e.g., Rodriguez et al., 2020a).

Most participants reported engagement in ongoing treatment, supporting the interpretation of PADs as complementary rather than substitutive interventions. The diversity of treatment pathways described (including medication, psychotherapy, and other specialist interventions) reflects a willingness to integrate PAD support alongside formal care. Participants frequently described PADs as enabling participation in everyday contexts, such as attending appointments and navigating public environments. In this way, PADs may facilitate ongoing engagement with treatment by helping individuals remain regulated and able to access care. This finding is supported by previous studies of psychiatric assistance dogs assisting both in terms of individual symptom relief (Smith et al., 2003) and as an important adjunctive form of treatment (Glintborg & Hansen, 2017).

Furthermore, these findings are consistent with prior research suggesting that assistance dogs may support engagement with treatment rather than displace it (Hansen et al., 2023). By extending these observations beyond veteran populations to a civilian sample, the present study broadens the applicability of existing evidence and suggests that the complementary role of PADs may be relevant across a wider range of mental health contexts. There is also a growing literature around extending the use of service dogs to a wider range of tasks (such as reminders about medication or assistance with sleep disturbance) and populations, including both younger and older adults with cognitive and psychiatric challenges (Audrestch et al., 2015; Monti et al., 2026; Mulraney et al., 2025; Salmon & Pachana, 2023).

In the current study, across participants’ accounts, safety emerged as the central dimension underpinning the perceived effectiveness of PADs. Rather than functioning solely as situational coping tools, PADs were frequently integrated into daily life as continuous sources of support. This was particularly evident in participants’ descriptions of PADs supporting task completion, trigger management, focus, and alerting in crowded or public places. Taken together, these findings suggest that PADs may operate as an external source of emotion regulation that supports functioning across everyday environments. Handlers described these supports as helping them remain oriented to the present, reduce distress responses, and maintain a sense of control in both public and emotionally demanding contexts. For some handlers, this constant presence positioned PADs as holistic support systems that combine elements of medication (constant availability), therapy (emotional regulation and grounding), and social support (facilitating participation in everyday activities). This conceptualisation extends prior research that has primarily emphasised task-based assistance or symptom interruption (Lloyd et al., 2019; O’Haire & Rodriguez, 2018) and highlights the relational and ongoing nature of PAD support.

The framing of PADs as holistic, integrated supports has important theoretical as well as practical implications. While safety and emotional stability were central benefits, sustained reliance on an external support system may also raise important questions about autonomy and long-term emotion regulation. Some researchers have cautioned that prolonged external reassurance seeking and soothing could, in certain contexts, impede the development of internal self-regulatory capacities (Starr et al., 2023). From this perspective, PAD use may involve a dynamic balance between external regulation and internal coping. Understanding how PADs interact with long-term emotion regulation, independence, and psychological resilience remains an important area for future research, particularly given the deeply embedded role PADs appear to play in handlers’ daily lives.

The degree to which many respondents relied on their PAD also raises important considerations, not only with respect to the independence of the handler, as mentioned above, but also in relation to the welfare of the animal. Guidelines about the working hours and the importance of downtime are essential ethical considerations in assistance animal welfare guidelines (Salmon et al., 2022). Some authors have raised concerns about the interaction of particular psychiatric symptoms and the ability to recognise welfare issues in PADs in a timely manner (Kiiroja et al., 2025). Assistance and therapy animals are agents in this exchange with humans, and inadvertent failures to recognise behavioural cues or welfare concerns remain understudied (Hatch, 2007). Given the conceptualisation of PADs as long-term, integrated support systems, organisational responses may also need to address lifecycle considerations such as working life guidelines, retirement, succession, and bereavement after loss of the PAD, which extend beyond the scope of initial training (Serpell et al., 2010). There is the beginning of literature addressing planning, loss and bereavement in relation to service animals that could and should be extended to PADs (Ng & Fine, 2019; Salmon et al., 2024).

Alongside perceived benefits, participants described a range of challenges that constrained the effectiveness of PAD support. These included public misunderstanding and stigma, legal uncertainty regarding access rights, and the financial burden associated with training and maintenance. Together, these challenges undermine the full potential of PADs to function as effective mental health supports and point to clear implications for policy design and broader systems of social support. Public misunderstanding and stigma may amplify psychological distress and limit participation in everyday activities, while legal ambiguity can create barriers to access in public spaces. Financial strain is particularly salient given the economic vulnerability observed among many handlers and may restrict the sustainability of PAD use over time. The qualitative themes further indicate that these barriers were experienced not only as practical inconveniences but as emotionally burdensome encounters that could increase anxiety, reinforce the visibility of disability, and undermine participants’ confidence in public settings. Our results support other researchers who point out issues with stigma in service animal use, particularly with hidden disabilities, and growing numbers of handlers are contributing their views on these issues (Audrestch et al., 2015; Laskin, 2023; McManus et al., 2021).

### 4.1. Implications

These findings suggest several directions for policy and practice. Targeted public education initiatives may help reduce stigma and inappropriate interference by improving societal understanding of PADs and their role. Public education should therefore address not only formal access rights but also everyday public behaviours such as questioning handlers, distracting or touching working dogs, and challenging the legitimacy of invisible disabilities. Clearer and more consistent access regulations could address ongoing legal barriers and reduce uncertainty for handlers. Financial support mechanisms, including expanded funding for PAD training and maintenance, may be particularly important for ensuring equitable access to this form of support.

Beyond formal policy frameworks, the findings also underscore the role of organisations as key components of the social support systems surrounding PAD use. While participants generally valued the training provided by organisations such as mindDog, training alone was often perceived as insufficient in the face of persistent social and structural barriers. These findings suggest that organisations may strengthen their impact not only by refining training protocols but also by engaging in public education, advocacy for legal reform, and the provision of ongoing psychosocial support for handlers.

Taken together, the findings of this study contribute to a growing body of literature that conceptualises PADs as more than task-oriented assistance animals. Instead, PADs appear to function as embedded, relational supports that shape daily functioning, emotional regulation, and social participation. By linking individual experiences with personal and systemic challenges, this study highlights the importance of aligning training practices, organisational support, and policy frameworks to enable PADs to safely realise their full potential as complementary mental health supports.

### 4.2. Limitations and Future Directions

Several limitations should be considered when interpreting these findings. First, participants were recruited from a single organisation and consisted of current PAD handlers who had successfully established partnerships with their dogs. As a result, the sample likely represents individuals for whom PAD use was maintained over time, and experiences of unsuccessful placement or early discontinuation were not captured. The findings therefore reflect how PADs function in established partnerships rather than the average outcome of PAD placement. In addition, the results should be understood as describing lived experience and perceived mechanisms of support, particularly in relation to safety and emotional regulation in everyday contexts.

An additional limitation concerns the absence of direct measures of animal welfare. Although the present study highlights the central role of PADs in supporting safety and emotional regulation, it did not directly assess the potential impact of these roles on the well-being of the dogs themselves. As PADs are active participants in these partnerships, this represents an important gap in understanding PAD use as a bi-directional system involving both human benefit and animal welfare.

A further limitation is the limited demographic and contextual information collected about both participants and their PADs. Although basic participant characteristics were reported, the survey did not capture more detailed information that may shape PAD use and experience, such as severity or duration of mental health conditions, functional impairment, or broader social and economic circumstances. In addition, limited information was collected about the dogs themselves, including age, breed, training history, working duration, or health status. Future research should collect more detailed handler and dog-level data to better understand how individual and dyadic characteristics influence PAD use, perceived effectiveness, and welfare outcomes.

Finally, this exploratory study relied on descriptive quantitative data integrated with qualitative accounts. This design does not allow causal conclusions about whether PADs directly produce improvements in mental health outcomes. Rather, the findings indicate how handlers interpret and use PAD support in daily life and how this support may interact with ongoing treatment and participation in community environments.

These limitations suggest several directions for future research. Future studies should adopt more comprehensive and integrative designs to better capture the complexity of PAD partnerships. Longitudinal approaches following handlers from pre-placement through different stages of partnership would help clarify how reliance on PADs develops over time and whether PAD presence influences engagement with treatment. Longitudinal studies in other areas of service dog use are expanding our understanding of such partnerships over time (e.g., Lundqvist et al., 2018) and would be instrumental in tracking how PAD reliance shifts over time. Expanding samples to include multiple organisations and a wider range of handler experiences, including unsuccessful placements, would further improve generalisability. Importantly, future research should also adopt a more integrated perspective by examining both human and animal outcomes. This may include the assessment of behavioural and physiological indicators of stress and well-being in PADs, contributing to a more comprehensive understanding of PAD partnerships as bi-directional systems involving both human benefit and animal welfare.

## 5. Conclusions

This study contributes to a growing body of literature by conceptualising psychiatric assistance dogs (PADs) as more than task-oriented interventions. The findings suggest that PADs may function as ongoing, relational supports embedded within everyday life, facilitating emotional regulation, perceived safety, and participation in daily activities. In this way, PADs may support engagement with mental health treatment by enabling individuals to remain regulated and able to access care, rather than replacing formal therapeutic interventions.

At a broader level, the effectiveness of PAD support appears to be shaped not only by individual characteristics but also by wider social and institutional contexts, including public understanding, legal frameworks, and financial accessibility. These findings highlight the importance of aligning training practices, organisational support, animal welfare considerations, and policy environments to support the sustainable integration of PADs within mental health care systems. Taken together, this study advances a more integrated perspective of PADs as complementary, context-embedded supports within complex systems of care.

## Data Availability

The data presented in this study are available on reasonable request from the corresponding author. The data are not publicly available due to privacy and ethical considerations.
